# Analysis of similarities and differences between transient symptomatic zinc deficiency and acrodermatitis enteropathica in children: a case report of a Chinese Yi-ethnic infant

**DOI:** 10.1186/s12887-024-04830-y

**Published:** 2024-05-16

**Authors:** Li Gu, Xue-Hui He, Peng Zhu

**Affiliations:** 1 Department of Pediatrics, Yibin Hospital Affiliated to Children’s Hospital of Chongqing Medical University , No. 108, Shangmao road, Xuzhou district, Yibin, Sichuan P.R. China; 2 Department of Pediatrics, the First People’s Hospital of Yibin, Yibin, Sichuan P.R. China

**Keywords:** Zinc deficiency, Aquired, Acrodermatitis enteropathica, *SLC39A4* gene, Case report

## Abstract

**Background:**

Transient symptomatic zinc deficiency (TSZD), an acquired type of zinc deficiency, is a rare, but probably underrecognized disease, extremely in breastfed premature with low birthweight infants. Its clinical manefestations are similar to Acrodermatitis enteropathica (AE), which is a genetic zinc absorption disorder caused by *SLC39A4* gene mutations. This gene encodes a member of the zinc/iron-regulated transporter-like protein (ZIP) family. The encoded protein localizes to cell membranes and is required for zinc uptake in the intestine. TSZD is often misdiagnosed as AE because of their extremely similar manefestations, characterized by a typical rash. Therefore, the differention between them is still a clinical challenging.

**Case presentation:**

Here, we present a case of TSZD in a 4 month and 23 days female Chinese Yi-ethnic premature with AE-like skin lesions, mainly presenting periorificial, perianal and perineal crusted, eroded, erythemato-squamous eruption. Laboratory examination showed the patient’s blood zinc level was significantly decreased. Further sequencing of the *SLC39A4* gene showed no mutation in the infant and her parents. Skin lesions significantly improved after 6 days of initial zinc supplementation (3 mg/kg/d), and maintenance treatment with 1 mg/kg/day of zinc was discontinued after 8 months without recurrence.

**Conclusions:**

The clinical manifestations of TSZD and AE are extremely similar, leading to a high rate of clinical misdiagnosis. While genetic analysis of the *SLC39A4* gene is a reliable method for differentiating TSZD from AE. It is recommended that *SLC39A4* gene test should be performed as far as possible in children with AE-like rash.

## Background

Zinc is a vital micronutrient for the human body. As an essential coenzyme in metalloenzymes, including alkaline phosphatase, it regulates gene expression and is an important structural component of gene regulatory proteins, such as those required for intracellular binding of tyrosine kinases to T cell receptors [1]. Zinc plays a variety of biological roles, including promoting growth and sexual organ development, maintaining taste and immune function, repairing skin and mucosa injury [2, 3].

Zinc deficiency is clinically classified as genetic and acquired. The genetic disease, known as acrodermatitis enteropathica (AE, OMIM #201,100), is a rare autosomal recessive disease due to a mutation of the *SLC39A4* gene (MIM #607,059), which encodes zinc/iron-regulated transporter-like protein 4 (ZIP4), leading to an impaired zinc absorption from the intestine [4, 5]. Transient symptomatic zinc deficiency (TSZD), the most common type of acquired zinc deficiency in infants, usually occurs in preterm, low birth weight and exclusively breastfed infants because of their negative zinc balance secondary to inadequate stores and intakes, malabsorption, increased requirements and possible low zinc level in the mother’s milk [6–8]. The clinical manifestations are similar in both inherited and acquired zinc deficiency characterized by dermatitis, alopecia and diarrhoea [9], so the differentiation is a big challenge. Severe zinc deficiency and the lack of zinc supplementation could lead to an increased risk of morbidity and mortality in young children. Thus, the early identification and treatment of this disorder should be considered as a medical emergency [9].

## Case description

A 4-month and 23-day-old Yi-ethnic girl was admitted to our hospital due to recurrent skin lesions with a 2-month history, accompanied by hair loss and intermittent mild diarrhea with 3–5 yellow, loose stools per day. Perioral erythema first appeared two months after her birth, and the rash gradually spread to the ears, occipital area, perianal and perineal regions, and distal extremities. She was treated as ecazema in local hospitals with external medications, such as Fusidic Acid Cream, Naristollae Scutellarin Compositus, but the therapeutic effect was poor. Simultaneously, due to the physician’s concerns that the baby’s diarrhea might be associated with cow’s milk protein allergy or lactose intolerance, breastfeeding was discontinued for a fortnight. However, the administration of specialized formula failed to ameliorate the rash and diarrhea during this period.The girl was born prematurely at 31 weeks (birth weight: 1540 g), hospitalized in the neonatology department for a duration of 33 days, receiving parenteral nutrition (PN) for 16 days, and was exclusively breastfed since discharge. In addition, the parents had a non-consanguineous marriage. This patient had no siblings and no similar manifestations had been observed in any other family members. As her mother did not exhibit any similar symptoms, her blood zinc levels were not tested.

**Fig. 1:**
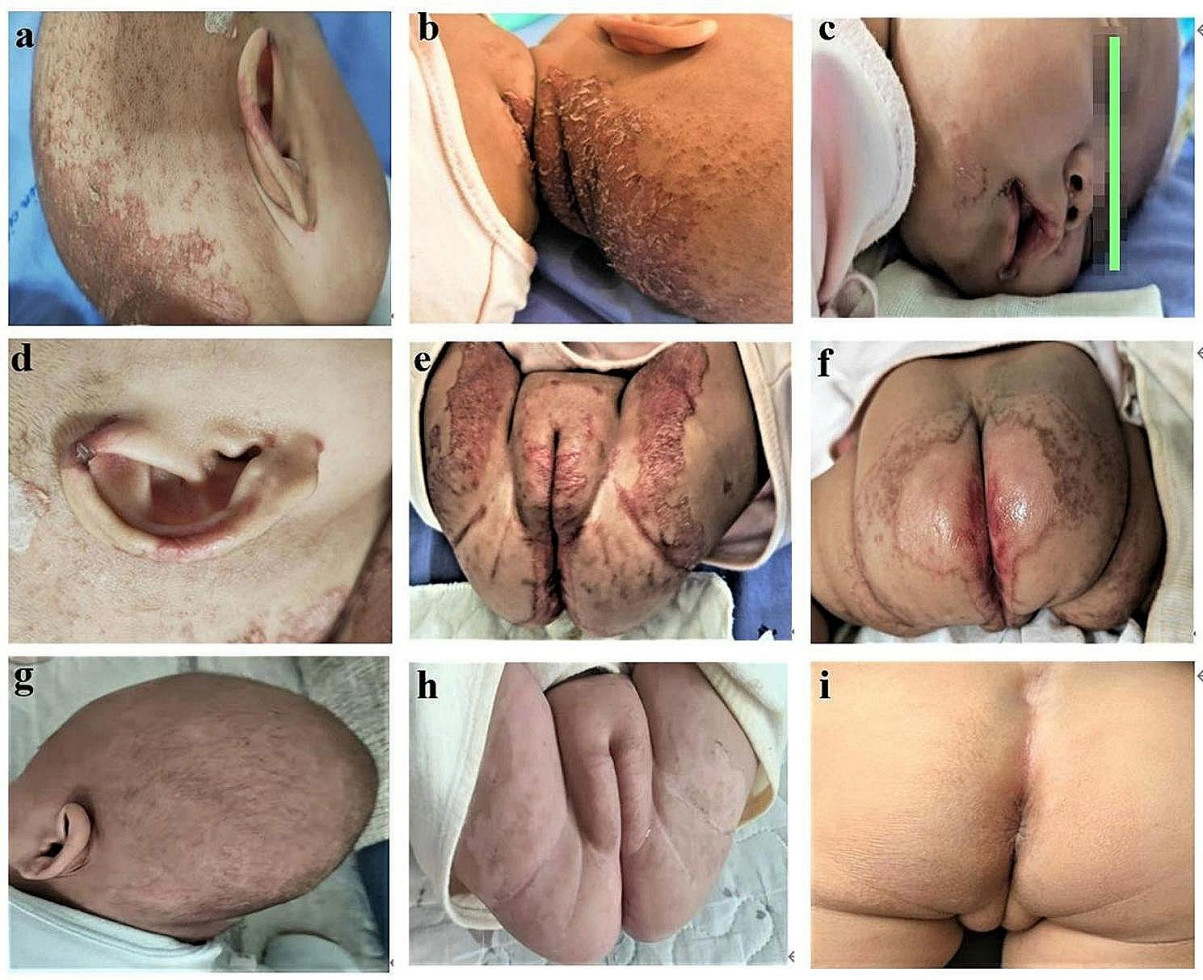
The patient had well-demarcated, scaly erythema, erosions and scabs on occipital area (**ab**), perioral area (**c**), ear (**d**), perineal area (**e**) and perianal area (**f**) before zinc supplementation. Please note diffuse alopecia on the scalp. The skin lesions were well improved after 6 days of treatment (**g**-**i**)

At the time of admission, the patient displayed signs of failure to thrive, with a weight of 5 kg (< 3rd percentile) and a length of 60 cm (< 10rd percentile). The dermatological examination revealed sparse hair and well-defined erythematous erosive plaques with scales and cursts at the periphery, primarily affecting the occipital and anogenital regions (Fig. 1a-f).

Laboratory and biochemical studies showed that the infant’s serum zinc levels were dramatically low with a value of 3.2 µmol/L (reference range 11.0–26.0 µmol/L). The serum albumin and alkaline phosphatase (ALP) values were normal. Additional tests includiing routine bloodwork, liver and kidney function assessments, autoimmune antibody profiles, lymphocyte immunophenotyping, and blood culture, all yielded negative results. The skin microscopic examination did not reveal any significant findings, other than the macroscopic skin lesions. Sanger sequencing demonstrated there was no mutation in the *SLC39A4* gene. Consequently, based on the clinical characteristics, the patient was diagnosed with TSZD.

The girl was initially administered an oral zinc supplementation of 3 mg/kg/day, which led to a significantly improvement in her skin lesions within three days and a complete resolution after sixdays (Fig. 1g-i), without anysigns of diarrhea. Serum zinc levels reached a normal value of 12.9 µmol/L after three days of zinc supplementation. After discharge, zinc supplementation was maintained for eight months at a dose of 1 mg/kg/d, and it was discontinued after weaning when the child turned one year old.To date, the girl had been followed up for 17 months. Although the girl’s growth and development remained poor, with a weight of 10 kg (10-25rd percentile) and a height of 75 cm (< 3rd percentile) due to the uniquedietary habits of her ethnic minority background, such as a two-meal system, a comparatively uniform food category and the tradition of mouth-to-mouth feeding for infants, and her picky eating, the AE-like skin lesions did not recur.

## Discussion and conclusion

Infants and children develop zinc deficiency when the demand exceeds its supply. Inherited zinc deficiency, known as AE, has an incidence rate of 1/500,000 [10]. The condition is due to the *SLC39A4* gene mutations, which limits the zinc absorption of the ZIP4 transporter in the small intestines, resulting in insufficient zinc absorption in the duodenum and jejunum [11]. Statistics have shown that the prevalent high-frequency mutations of AE are located in exons 9, 3 and 5, and the clinical phenotypes vary with different exron mutations [12]. Periorificial dermatitis, alopecia and diarrhoea are the characteristic symptoms of AE, but these three symptoms simultaneously occur in only 20% of patients [13]. The systemic features also encompass irritability, lethargy, anorexia, growth impairment, anemia, neuropsychiatric disorders, immunological aberrations, and ocular anomalies such as conjunctivitis, blepharitis, corneal opacities, and photophobia [14].

TSZD, the acquired form, is a self-limited disease primarily observed in exclusively or partially breastfed premature and low birth weight infants[15–17, 8], but it has also been reported in full-term infants [18–20, 7]. Compared to AE, the etiologies of TSZD are more diverse, including preterm, low birth weight, PN history andothers. Preterm and low birth weight infantsare more susceptible to developing zinc deficiency than full-term infants due to their inadequate zinc stores, compromised ability to absorb zinc from the intestine and increased zinc requirements during catch-up growth [21]. PN is considered a risk factor of acquired zinc deficiency in numerousreports, associated with a lack of awareness and inadequate management of the importance of trace elements in PN, leading to an iatrogenic TSZD [22, 23]. In the case we report, the simultaneous presence of aforementioned three risk factors -preterm, low birth weight, and a history of PN- result in severe zinc deficiency shortly after birth. It is worth noting that an increasing number of case reports on TSZD in full-term infants have been documented in recent years. This suggests that besides factors such as preterm birth, other mechanisms are involved in the pathogenesis of TSZD. Studies demonstrate that *SLC30A2* gene mutations of nusing monthers lead to low zinc levels in breast milk, which serves as one of the causes of TSZD in full-term infants who areexclusively or partially breastfed [6]. *SLC30A2* gene mutations could result in dysfunction of the zinc transporter 2 (ZnT2), which is responsible for zinc secretion into breast milk [24, 25]. Unfortunately, one limitation of this case is that we are unable to assess the zinc levels in the mother’s milk, due to the unique customs of the ethnic minorities involved. We did not further encourage the mother to undergo testing for the *SLC30A2* gene in consideration of the financial challenges faced by the family. The reason is that the absence of any amelioration in rash or diarrhea symptoms during the two-week period of breastfeeding discontinuation in this case led us to presume that the zinc levels in breast milk are not low. Provided that the mother’s *SLC30A2* gene mutations result in decreased zinc levels in breast milk, and subsequently leads to insufficient zinc intake in exclusively breastfed or breastfed infants exhibiting zinc deficiency symptoms, it is anticipated that the symptoms will experience significant improvement following the cessation of breastfeeding. However, this is not the case. Currently, the exact prevalence of TSZD remains obscure, potentially underestimated. In a Chinese research study, 18 children were initially diagnosed with AE based on the typical skin lesions and low blood zinc levels. However, no mutation was detected in *SLC39A4* sequencing outcomes. In conjunction with the clinical manifestations, strong response to zinc supplementation, and absence of recurrence following cessation of zinc therapy, these patients were ultimately diagnosed with TSZD [26]. Thus, it can be seen that the recognition of TSZD is insufficient and the important value of *SLC39A4* gene test in the differential diagnosis between TSZD and AE is overlooked. Although the clinical manifestations of TSZD and AE are alike, there are distinctions in the onset and prognosis. TSZD typically occurs during breastfeeding, necessitates only short-time zinc supplementation and dosenot recur after stopping zinc treatment.Conversely, AE predominantly presents after weaning, necessitates lifelong zinc supplementation, and is susceptible to relapse when zinc supplementation isceased. It is interesting to note that there is currently no literature on the definite time of zinc supplementation for childern with TSZD. It is observed that the treatment duration reported in the literature ranged from less than a month to over 22 months [27, 28]. Therefore, we deem that the short-term zinc supplementation of TSZD is a relative concept in the context of lifelong zinc supplementation of AE.

Collectively, we report a case with AE-like skin lesions. Based on a comprehensive evaluation of characteristics such as preterm birth, low birth weight, a history of PN, hypozincemia, the absence of *SLC39A4* gene mutations, a marked response to zinc therapy, and the absence of recurrence following the withdrawal of zinc supplementation, the premature infant was ultimately diagnosed with TSZD. By reporting this case, we emphasize the challenges associated with distinguishing TSZD from AE, and recommend that routine testing for the *SLC39A4* gene be performed for patients with AE-like dermatitis. When the *SLC39A4* gene test is not conducted, we underscore the necessity of a long-term follow-up to observe whether zinc deficiency-related symptoms reappeared after zinc discontinuation, in order to draw definitive diagnostic conclusions.

## Data Availability

The datasets presented in this article are not readily available because of ethical and privacy restrictions. Requests to access the datasets should be directed to the corresponding author.

## References

[1] Ogawa Y, Kinoshita M, Shimada S, Kawamura T (2018). Zinc and skin disorders. Nutrients.

[2] Hammersen J, Has C, Galiano M, Lindner M, Rossi R, Kohlhase J (2018). Sustained need for high-dose zinc supplementation in children with Acrodermatitis Enteropathica. Clin Pediatr (Phila).

[3] Thirumoorthy N, Shyam Sunder A, Manisenthil Kumar K, Senthil Kumar M, Ganesh G, Chatterjee M (2011). A review of metallothionein isoforms and their role in pathophysiology. World J Surg Oncol.

[4] Kasana S, Din J, Maret W (2015). Genetic causes and gene–nutrient interactions in mammalian zinc deficiencies: acrodermatitis enteropathica and transient neonatal zinc deficiency as examples. J Trace Elem Med Biol.

[5] Küry S, Dréno B, Bézieau S, Giraudet S, Kharfi M, Kamoun R (2002). Identification of SLC39A4, a gene involved in acrodermatitis enteropathica. Nat Genet.

[6] Yang WL, Hsu CK, Chao SC, Huang CY, Lee JYY (2012). Transient zinc deficiency syndrome in a breast-fed infant due to decreased zinc in breast milk (type II hypozuncemia of infancy): a case report and review of the literature. Dermatol Sin.

[7] El Fékih N, Monia K, Schmitt S, Dorbani I, Küry S, Kamoun MR (2011). Transient symptomatic zinc deficiency in a breast-fed infant: relevance of a genetic study. Nutrition.

[8] Barruscotti S, Vassallo C, Giorgini C, Savasta S, Licari A, Marseglia GL (2019). Transient symptomatic zinc deficiency in a breast-fed African infant: case report and literature review. Int J Dermatol.

[9] Glutsch V, Hamm H, Goebeler M (2018). Zinc and skin: an update. JDDG.

[10] Nakano H, Nakamura Y, Kawamura T, Shibagaki N, Matsue H, Aizu T (2009). Novel and recurrent nonsense mutation of the SLC39A4 gene in Japanese patients with acrodermatitis enteropathica. Br J Dermatol.

[11] Kilic SS, Giraud M, Schmitt S, Bézieau S, Küry S (2007). A novel mutation of the SLC39A4 gene causing acrodermatitis enteropathica. Br J Dermatol.

[12] Zhong W, Yang C, Zhu L, Huang YQ, Chen YF (2020). Analysis of the relationship between the mutation site of the SLC39A4 gene and acrodermatitis enteropathica by reporting a rare Chinese twin: a case report and review of the literature. BMC Pediatr.

[13] Garza-Rodríguez V, de la Fuente-García A, Liy-Wong C, Küry S, Schmitt S, Jamall IS (2015). Acrodermatitis Enteropathica: a novel SLC39A4 gene mutation in a patient with normal zinc levels. Pediatr Dermatol.

[14] Martínez-Bustamante ME, Peña-Vélez R, Almanza-Miranda E Acrodermatitis enteropática [Acrodermatitis enteropathica]. Bol Med Hosp Infant Mex., Amico G, De LC, Smits G, Salik D, Deprez G, Vilain C et al. Acquired Zinc Deficiency Mimicking Acrodermatitis Enteropathica in a Breast-Fed Premature Infant. Pediatr Rep. 2021;13(3):444-9. doi:10.3390/pediatric13030051. 10.3390/pediatric13030051 PMC8396245 34449696

[16] Vashist S, Rana A, Mahajan VK (2020). Transient symptomatic Zinc Deficiency in a Breastfed Infant Associated with low zinc levels in maternal serum and breast milk improving after zinc supplementation: an uncommon phenotype?. Indian Dermatol Online J.

[17] Kiechl-Kohlendorfer U, Fink FM, Steichen-Gersdorf E (2007). Transient symptomatic zinc deficiency in a breast-fed preterm infant. Pediatr Dermatol.

[18] He Y, Yang Q, Pradhan S, Ran Y, Wang S (2021). Transient symptomatic Zinc Deficiency Resembling Acrodermatitis Enteropathica in a full-term Breastfed Infant. Indian J Pediatr.

[19] Crisóstomo M, Santos MC, Tavares E, Cunha F (2021). Transient symptomatic zinc deficiency in an exclusively breastfed infant. BMJ Case Rep.

[20] Dassoni F, Abebe Z, Ricceri F, Morrone A, Albertin C, Naafs B (2014). High frequency of symptomatic zinc deficiency in infants in northern Ethiopia. Dermatol Res Pract.

[21] Al-Mendalawi MD (2022). Transient symptomatic Zinc Deficiency: an overlooked diagnosis in Acrodermatitis Enteropathica like Eruption in an exclusively breastfed Preterm Infant. Oman Med J.

[22] Wiznia LE, Bhansali S, Brinster N, Al-Qaqaa YM, Orlow SJ, Oza V (2019). Acquired Acrodermatitis Enteropathica due to zinc-depleted parenteral nutrition. Pediatr Dermatol.

[23] Livingstone C, Zinc (2015). Physiology, deficiency, and parenteral nutrition. Nutr Clin Pract.

[24] Muto T, Kawase Y, Aiba K, Okuma M, Itsumura N, Luo S (2023). Novel SLC30A2 mutations in the pathogenesis of transient neonatal zinc deficiency. Pediatr Investig.

[25] Itsumura N, Kibihara Y, Fukue K, Miyata A, Fukushima K, Tamagawa-Mineoka R (2016). Novel mutations in SLC30A2 involved in the pathogenesis of transient neonatal zinc deficiency. Pediatr Res.

[26] Wang S, Yang Y (2014). Clinical analysis of 18 cases of transient symptomatic zinc deficiency and SLC39A4 gene detection. J Clin Dermatol.

[27] Connors TJ, Czarnecki DB, Haskett M (1983). Acquired zinc deficiency in a breast-fed premature infant. Arch Dermatol.

[28] Coelho S, Fernandes B, Rodrigues F (2006). Transient zinc deficiency ina breast fed, premature infant. Eur J Dermatol.

